# Pervasive promotion of breastmilk substitutes in Phnom Penh, Cambodia, and high usage by mothers for infant and young child feeding

**DOI:** 10.1111/mcn.12271

**Published:** 2016-04-15

**Authors:** Alissa M. Pries, Sandra L. Huffman, Khin Mengkheang, Hou Kroeun, Mary Champeny, Margarette Roberts, Elizabeth Zehner

**Affiliations:** ^1^ Helen Keller International Asia Pacific Regional Office Phnom Penh Cambodia; ^2^ Consultant to Helen Keller International; ^3^ Helen Keller International Phnom Penh Cambodia; ^4^ Helen Keller International Washington D.C. USA

**Keywords:** breastfeeding, breastmilk substitutes, Cambodia, promotion, prelacteal feedinginfant feeding, infant feeding

## Abstract

In 2005, Cambodia passed the *Sub‐Decree on Marketing of Products for Infant and Young Child Feeding* (no. 133) to regulate promotion of commercial infant and young child food products, including breastmilk substitutes. Helen Keller International assessed mothers' exposure to commercial promotions for breastmilk substitutes and use of these products through a cross‐sectional survey among 294 mothers of children less than 24 months of age. Eighty‐six per cent of mothers reported observing commercial promotions for breastmilk substitutes, 19.0% reported observing infant and young child food product brands/logos on health facility equipment and 18.4% reported receiving a recommendation from a health professional to use a breastmilk substitute. Consumption of breastmilk substitutes was high, occurring among 43.1% of children 0–5 months and 29.3% of children 6–23 months of age. Findings also indicated a need to improve breastfeeding practices among Phnom Penh mothers. Only 36.1% of infants 0–5 months of age were exclusively breastfed, and 12.5% of children 20–23 months of age were still breastfed. Children that received a breastmilk substitute as a prelacteal feed were 3.9 times more likely to be currently consuming a breastmilk substitute than those who did not. Despite restriction of commercial promotions for breastmilk substitutes without government approval, occurrence of promotions is high and use is common among Phnom Penh mothers. In a country with high rates of child malnutrition and pervasive promotions in spite of restrictive national law, full implementation of Cambodia's Sub‐Decree 133 is necessary, as are policies and interventions to support exclusive and continued breastfeeding.

**Key messages**

- Despite prohibition without specific approval by the national government, companies are pervasively promoting breast‐milk substitutes in Phnom Penh, particularly on television and at points of sale.
- Strengthened implementation and enforcement of Cambodia's subdecree 133 are needed to better regulate promotion in order to protect breastfeeding for the nutrition and health of infants and young children in Cambodia.
- Mothers who used a breast‐milk substitute as a prelacteal feed were 3.9 times more likely to currently feed this same child a breast‐milk substitute, as compared with mothers who did not provide breast‐milk substitute as a prelacteal feed. Supporting breastfeeding among mothers after delivery is critical to establish and sustain optimal breastfeeding practices.
- Use of breast‐milk substitutes is also very common among mothers of children under 2 years of age in Phnom Penh. We recommend promoting exclusive and continued breastfeeding as beneficial to children's health and development, and supporting policy and workplace environments that enable breastfeeding up to and beyond 24 months of age.

## Introduction

Exclusive breastfeeding for the first 6 months of life and continued breastfeeding up to 2 years of age or beyond are recommended to ensure optimal child growth and development [World Health Organization (WHO) & UNICEF 2003]. Childhood undernutrition remains a serious concern in Cambodia. Thirty‐two per cent of children less than 5 years of age are stunted, 24% are underweight, 10% are wasted, and 56% are aneamic (NIS 2015). The 2014 Cambodian Demographic and Health Survey (CDHS) found that the percentage of children stunted increases with age, from 16% among children less than 6 months of age to 17% among children aged 9–11 months and to 34% between 18 and 23 months of age (NIS 2015).

In 2009, the Cambodia Socioeconomic Survey found that 98% of Cambodian women reported having ever breastfed their youngest child (NIS 2009). Exclusive breastfeeding rates among infants less than 6 months of age increased between 2000 and 2010 from 11% to 74% but declined to 65% in 2014 (NIS 2015). A study by Prak *et al.* notes that this initial progress in breastfeeding practices may be due to the advent of infant and young child feeding (IYCF)‐focused public health campaigns; however, the study also notes the increasing use of breastmilk substitutes across Cambodia that could curtail these efforts (Prak *et al*. 2014). In 2010, 4.7% of currently breastfed and 25.5% of nonbreastfed children below 24 months of age were being fed breastmilk substitutes (NIS 2011). Bottle use is also increasing, especially among older infants. In 2005, 11.8% of newborns aged 12–23 months were fed with a bottle and nipple, compared with 24.7% of children in 2010 (NIPH/NIS 2006; NIS 2011).

In response to documented unethical marketing activities by breastmilk substitute manufacturers, the WHO developed the *International Code of Marketing of Breast‐milk Substitutes* (WHO international code) and subsequent resolutions. The Cambodian government adopted much of this code as a national policy in the Cambodian Sub‐Decree *on Marketing of Products for Infant and Young Child Feeding* (no. 133, November 2005), which aims to promote and support breastfeeding by regulating the promotion of commercial food products, including breastmilk substitutes and complementary foods, marketed for children less than 2 years of age. The subsequent *Joint Prakas on the Marketing of Products for Infant and Young Child Feeding* (no. 061, August 2007) is intended to operationalize the implementation and monitoring of the Sub‐Decree 133. In August 2014, 6 months after the culmination of this study, the national government established the oversight board for the implementation of the Sub‐Decree 133 and Joint Prakas, bringing together the Ministry of Health and other relevant line ministries. Until now, however, oversight board monitoring of promotions has not yet been implemented.

Information regarding the messages that mothers in Phnom Penh report receiving from the health system and the commercial sector about IYCF can inform efforts to reinforce positive messages about breastfeeding and to prevent breastmilk substitute promotion. Numerous studies have used interviews with mothers to assess the promotion of breastmilk substitutes and/or IYCF counselling received through the health system (IGBM & UNICEF 2005; Save the Children & Gallup Pakistan 2013; Taylor 1998; Aguayo *et al*. 2003; Hamilton 2002; Sobel *et al*. 2011; Haiek 2012; Babak *et al*. 2004; Brownlee 2009; Perez‐Escamilla 2004). These studies have documented the promotion of breastmilk substitutes within health systems, including the provision of free samples and the presence of promotional materials, as well as the point of sale promotions, which are all prohibited by the *International Code of Marketing of Breast‐milk Substitutes* (WHO 1981). In the 2004 Perez‐Escamilla study, this information was fed back to health centre staff and led to improvements in health system practices.

This study assessed mothers' exposure to commercial promotions for breastmilk substitutes in Phnom Penh, Cambodia, and their use of these products. Specifically, the objectives of the research were as follows: (1) to estimate exposure to promotional practices occurring within and outside the health system for breastmilk substitutes, including infant formula, follow‐on formula and growing‐up/toddler milks; (2) to document breastfeeding support and counselling provided in health facilities; and (3) to document the consumption of breastmilk substitutes among infants and young children in Phnom Penh, Cambodia.

## Materials and methods

This cross‐sectional survey among mothers of children less than 24 months of age residing in Phnom Penh, Cambodia used a multistage sampling procedure to obtain a representative sample. Because variables of interest included breastfeeding practices, the study was limited to only mothers and did not include other caregivers of children. Recruitment of participants and data collection were conducted at health facilities, with this population serving as a proxy for sampling mothers from the general population. Sampling of mothers stratified children across age categories to allow for an equal age distribution that would be representative of the general Phnom Penh population. From November 2013 to February 2014, researchers collected data through structured interviews, in which they asked mothers of children less than 24 months of age to recall exposure to promotions since the birth of their youngest child and to describe current feeding practices for this same child.

Because of concern for high rates of consumption of commercial IYCF products, especially in urban areas compared with rural (Huffman *et al* 2014), we limited this survey to mothers currently living in and using health facilities within Phnom Penh. Researchers excluded mothers with any characteristics that can delay breastfeeding initiation [mothers of newborns with congenital diseases or who were in the neonatal intensive care unit, mothers who experienced severe delivery complications during the birth of their newborn, mothers whose newborn is a twin or from a multiple birth and mothers with newborns too ill to proceed with an interview].

### Sample size

We calculated the sample size to detect an estimated 10% prevalence rate of exposure to promotions within the health system, with a measurement error of ±5%. Using a standard of error of 0.0255 and assuming a design effect of 2 to account for the cluster design, we determined a sample size of 280 mothers. Because of the cluster sampling design used (described later in the text), the final sample size was slightly higher than 280.

A total of 498 mothers using child health services at a facility were approached for interview. Ninety one (18.3%) of these 498 mothers refused participation, and 113 (22.7%) mothers were excluded; 96 (19.3%) mothers lived outside of Phnom Penh, 11 (2.2%) mothers reported severe complications during delivery, seven (1.4%) infants had been in the neonatal intensive care unit after delivery, and one (0.2%) child was from a multiple birth (mothers could have been excluded on more than one criterion). The majority of refusals by mothers were due to the mothers not having time and needing to leave the health facility after their child received services. Interviewers successfully completed interviews with 294 mothers with children under 24 months of age who were using child health services.

### Sampling procedure and data collection

Because of inability to obtain facility approval from private facilities, we sampled public facilities for the interviews. We obtained the lists of all public health facilities offering maternity and/or child health services from the Cambodian Ministry of Health. This included national hospitals, referral hospitals and health centres; health posts were excluded. In addition, utilisation rates for these facilities for the preceding year (2012) were also obtained, which included the total number of child health visits, including outpatient department and immunisation. We then calculated the annual rates as the monthly average per facility by dividing by 12 months. In order to complete data collection within the scheduled timeframe, we excluded facilities with less than 50 child health visits per month from the sampling frame. This eliminated six out of 37 public child health facilities, but the 31 included in the sampling frame represented 97.3% of all child health visits in Phnom Penh public health facilities.

**Figure 1:**
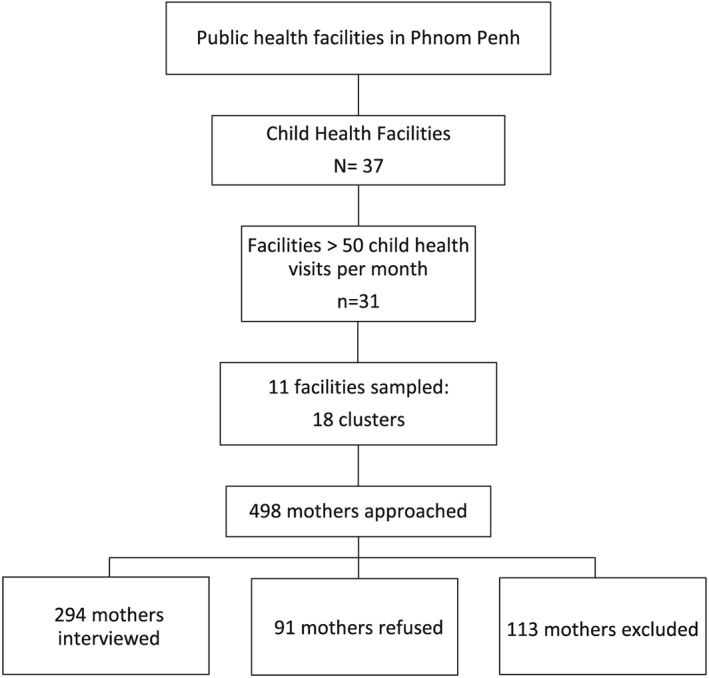
Sampling profile for mothers and facilities.

We then sampled all health facilities by allocating clusters using probability proportional to size. The calculated monthly utilisation rates served as each facility's or group of facilities' ‘population’. We assigned 18 clusters of 16 mothers each across facilities in the sampling frame; we chose the total 16 mothers per cluster to allow for even distribution of child ages across four age categories (0–5.9, 6–11.9, 12–17.9 and 18–23.9 months). Because sampling of facilities was proportional to size, larger facilities had a greater chance of being sampled for multiple clusters, while smaller facilities had a greater chance of being sampled for only one cluster. Eleven public health facilities were sampled. Fig. 1 details the sampling of facilities and mothers.

We alerted staff at sampled facilities about data collection approximately 1 week prior to survey. Survey supervisors approached women with children for interview at clinics offering child health services, either in the immunisation or in the outpatient department, at a sampled health facility. Survey supervisors screened every woman with a child who passed through the entrance/exit point of the child health clinic area. They first screened women to assess the following: (1) if they were the mother of the child with them; (2) if this child was under 24 months of age; and (3) if they lived in Phnom Penh. Survey supervisors also assessed the age of the child to verify if an interview was still needed for the specific age category. We obtained approval for this study from the Cambodia National Ethics Committee for Health Research prior to data collection 1Approval was obtained on 28 August 2013 (registration no. 0155/2013). and obtained informed consent from all participants prior to conducting interviews.

### Questionnaire design

The questionnaire collected data on mothers' characteristics, including age, marital status, educational attainment, household assets and drinking water source, and details regarding antenatal care (ANC) and delivery of the youngest child. Data collected specifically on the youngest child included age, gender and birth order. The interviewers also collected data on early and current breastfeeding practices on the preceding day for the youngest child, in accordance with the WHO guidelines on IYCF practices (WHO 2008). They used standardised questionnaires to obtain information on which foods and liquids were consumed by the youngest child on the day and night prior to the day of interview. Additionally, they gathered data on the types of foods mothers aspired to feed their youngest child and reasons why. They asked mothers to report on commercial promotional practices experienced inside and outside the health system since the delivery of their youngest child.

Interviewers used a mobile data collection system to allow for immediate data entry, reduction in data errors and prompt analyses. The research team designed the questionnaires in microsoft word (Microsoft, Redmond, Washington, USA) and then entered them in formhub, an open‐source online platform that enables data collection via phones or tablets, using the Android application Open Data Kit Collect. They submitted the data online to a web‐based database (Formhub.org 2013). The questionnaires were translated from English into Khmer, back translated into English to ensure accuracy and uploaded into formhub in Khmer. Interviewers conducted interviews in Khmer using the Samsung (Samsung, Seoul, South Korea) Galaxy tab 2.0 7″ model tablet. The research team reviewed the submitted questionnaires weekly to ensure data quality.

### Study definitions

This study defined breastmilk substitutes to include infant/starter formula (indicated for use from birth to 5 months of age), follow‐up formula (indicated for use from 6 to 11 months), infant/follow‐up formula for special dietary or medical purposes, growing‐up milk (indicated for use from 12 to 36 months) and other milk or milk‐like products (in liquid or powdered form) marketed or otherwise represented as suitable for feeding children younger than 2 years of age (WHO 1981). Mothers were also asked to report the brands of breastmilk substitutes mentioned in order to verify that these products fit this definition.

The study defined commercial promotions as any type of marketing technique intended to increase sales, including media or print advertising, provision of free samples or any other activities to encourage or induce the purchase of a product (International Baby Food Action Network 2007). It measured exposure to such commercial promotion within the health system by asking mothers if they had heard, seen or read any promotions during pregnancy or since the delivery of their newborns, and if so, where. Interviewers also asked mothers if they had received any free samples of products, and if so, where and from whom. Additionally, they asked mothers if they had observed any display of logos or brands on equipment or materials within a health facility. The study defined interpersonal promotions as recommendations/advice from a health professional to use a breastmilk substitute; we obtained the measurement of exposure to interpersonal promotion by asking mothers if they had received such a recommendation during pregnancy or since the delivery of their newborns. We defined and measured the use of breastmilk substitutes as a prelacteal feed, by asking mothers to report if they fed breastmilk substitute within the first 3 days after delivery, and current use of breastmilk substitutes was defined and measured by asking mothers if they had fed their youngest child a breastmilk substitute on the day prior to interview.

### Statistical analyses

Data were cleaned and analysed using spss version 21 (IBM, Armonk, NY, USA). Proportions for categorical and mean ± standard deviation for continuous variables were used to describe the sampled mothers. Differences in predictive variables (such as child age categories) by outcome variables (such as use of breastmilk substitutes) were assessed through comparisons using Pearson chi square to test for significance. Multivariate logistic regression was conducted to assess the impact of variables shown to influence children's current consumption of breastmilk substitutes during bivariate analysis. Covariates included in the model were child's age, maternal attainment of secondary or higher level of education, maternal working status and mother's use of breastmilk substitutes as a prelacteal feed for the youngest child. The model was also adjusted for cluster sampling. Goodness of fit for this model was determined through the Hosmer–Lemeshow test, with a significance of *P* = 0.110.

## Results

Demographic and socioeconomic characteristics for mothers of children less than 24 months of age visiting child health services are shown in Table 1. The majority of all mothers were currently married at the time of interview, and approximately half of mothers (52.7%, *n* = 155) reported their child under 24 months of age to be their only child. Among those who were currently married, 4.8% (*n* = 14) of mothers reported that their husband currently lived away from home. Over 90% of mothers (93.2%, *n* = 274) had attended any level of formal education, and 7.5% (*n* = 22) of mothers reported attending university or higher graduate studies. Approximately one‐fourth (24.1%, *n* = 71) of mothers visiting child health clinics reported currently working outside the home, and 83.0% (*n* = 244) reported themselves to be the main caregiver of their youngest child.

**Table 1:** Demographic and socioeconomic characteristics of mothers of children under 24 months of age

	Mothers with children < 24 months (*n* = 294)
Mother	
Age (years) (mean ± SD)	27.9 ± 4.9
Parity (number) (mean ± SD)	1.7 ± 1.0
Marital status (%)	
Married	98.6
Divorced, widowed or separated	1.4
Level of education (%)	
None	6.8
Nonformal education	0.0
Primary	35.4
Lower secondary	34.7
Upper secondary	15.6
Tertiary education	7.5
Works outside the home (%)	24.1
Main caregiver of child (%)	83.0
Received antenatal care (%)	92.9
Assisted delivery (%)	99.3
Child	
Age (mean ± SD) (months)	11.7 ± 6.7 (months)
0–5	24.5
6–11	24.8
12–17	24.5
18–23	26.2
Sex (female) (%)	46.6
C‐section delivery (%)	14.6
Household	
Safe source of drinking water (%)	95.2
Assets, ownership (%)	
Bicycle	39.1
Car	20.4
Motorbike	80.6
Refrigerator	25.9
Television	85.0

SD, standard deviation; C‐section, caesarean section.

The mean age of children less than 24 months of age was 11.7 months, as would be anticipated given the effort made to sample children across an equal distribution of ages 0–23 months. The majority (92.9%, *n* = 273) of mothers had received ANC during pregnancy with their youngest child. Fifteen per cent (*n* = 43) of children under 24 months of age were delivered via caesarean section, and almost all mothers (99.3%, *n* = 292) delivered their youngest child with the assistance of a health professional, including a doctor, nurse or auxiliary nurse midwife.

Almost all mothers reported a safe source of drinking water for their household, which included piped water, water from a tubewell, borehole or protected spring and commercial bottled water. The majority of mothers reported that their household owned a television and motorbike [85.0% (*n* = 250) and 80.6% (*n* = 237), respectively]. One‐fifth (20.4%, *n* = 60) of mothers reported that their household owned a car.

### Antenatal care and breastfeeding counselling

Mothers were asked about their exposure to breastfeeding counselling; results are shown in Table 2. Overall, just over half (56.5%, *n* = 166) of mothers received breastfeeding advice during ANC during pregnancy with their youngest child. The most commonly reported breastfeeding messages received among mothers were related to the promotion of exclusive breastfeeding and early initiation of breastfeeding. Information regarding the risks of feeding infant formula was reported by 8.8% (*n* = 26) of all mothers. Just over half of mothers (52.0%, *n* = 153) reported receiving assistance in positioning and/or attachment for breastfeeding from a health worker after delivery of their youngest child.

**Table 2:** Percentage of mothers who received breastfeeding messaging during antenatal care

	% of mothers (*n* = 294)
Receiving information on breastfeeding from a health worker during ANC (%)	56.5
Breastfeeding messages received during ANC	
Exclusive breastfeeding	32.3
Early initiation	30.3
Risks of feeding infant formula	8.8
Continued breastfeeding until 2 years and beyond	4.4
Risks of feeding other foods/liquids before 6 months	3.7
Hygiene during breastfeeding	2.4

ANC, antenatal care.

### Commercial promotion of breastmilk substitutes within the health system

Results regarding mothers' exposure to commercial promotions for breastmilk substitutes within the health system are shown in Table 3. Eighteen per cent of mothers (*n* = 54) received a recommendation from a health professional to use a breastmilk substitute since the birth of their youngest child; 4.1% of mothers reported receiving a recommendation to use product manufactured by Dumex, and 3.4% reported receiving a recommendation to use a France Bebe product. Mothers reported nurses and midwives as the most common sources of these health professional recommendations. Observation of commercial advertisements for breastmilk substitutes within the health system was not common among mothers interviewed; 11 mothers (3.7%) reported seeing, hearing or reading such an advertisement within a health facility since the birth of their youngest child. Nineteen per cent (*n* = 56) of mothers reported observing a breastmilk substitute or commercially produced complementary food brand or logo on health facility equipment or materials. Mothers reported the majority of branding observations on health facility posters. Eight per cent (*n* = 23) of mothers reported receiving a free sample of a breastmilk substitute from a health professional; just over half (52.2%) of these samples came from midwives, 26.1% were given by nurses, and 17.4% were provided by doctors (mothers were not able to identify the type of health professional for 4.3% of samples). Nine mothers (3.1%) also reported receiving a sample of a bottle or teat from a health professional. Two mothers (0.7%) reported receiving a gift of ‘mothers' milk’ from a health professional since the birth of their youngest child.

**Table 3:** Percentage of mothers exposed to commercial promotions for breastmilk substitutes within the health system

	% of mothers (*n* = 294)
Received recommendation to use breastmilk substitute from a health professional	18.4
Observed branding/logos on health facility equipment	19.0
Observed commercial advertisement of breastmilk substitute within health facility	3.7
Received breastmilk substitute sample from a health professional	7.8
Received bottle or teat sample from a health professional	3.1
Received a gift from a health professional branded with breastmilk substitute company	0.7

### Recommendations and commercial promotion outside the health system

Aside from health professionals, the most commonly reported sources of recommendations for breastmilk substitutes were personal contacts. Twenty per cent (*n* = 58) of mothers reported receiving a recommendation from a relative, and 14.3% (*n* = 42) reported receiving a recommendation from a friend or neighbour. Recommendations from commercial sector sources, such as company representatives or store owners, were rare. Recommendations to use a breastmilk substitute from any of these sources were reported by half of all mothers (50.3%, *n* = 148).

**Figure 2:**
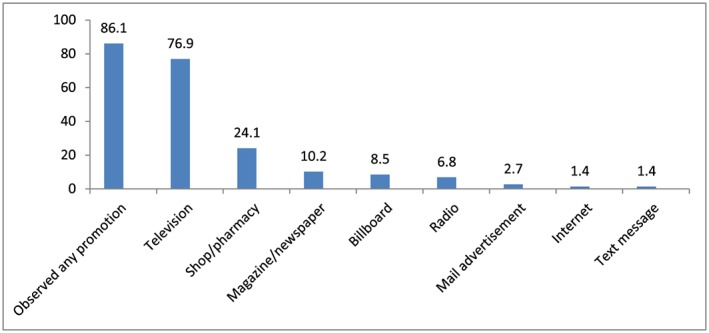
Percentage of mothers exposed to commercial promotion for breastmilk substitutes.

Interviewers asked mothers of children less than 24 months of age to recall if they had seen, heard or read a commercial promotion for infant and young child (IYC) food products since the birth of their youngest child (Fig. 2). Overall, 86.1% (*n* = 253) of mothers reported observing a breastmilk substitute promotion. Promotions for Dumex and France Bebe breastmilk substitute products were most common, being reported by 61.2% and 35.4% of mothers, respectively. Television (76.9%) was the most commonly reported source of promotions, while promotions in shops or pharmacies (24.1%) were the second most commonly reported location.

Reports of receipt of free samples, discounts or coupons for breastmilk substitutes outside the health system were rare. Only 14 (4.8%) of mothers reported receiving a free sample of a breastmilk substitute from a source outside the health system (10 from company representatives and four from a shop salesperson/pharmacist), and only 12 mothers (4.1%) reported receiving a discount or coupon for breastmilk substitutes.

### Feeding practices

The study assessed current breastfeeding practices for the youngest child; results are shown in Table 4. Almost all (97.3%, *n* = 286) children less than 24 months of age had ever been breastfed. Continued breastfeeding at 1 and 2 years of age was low; two‐thirds (67.3%) of mothers of children 12–15 months of age reported that their child was still breastfeeding, and only 12.5% of children 20–23 months of age were currently breastfeeding. Exclusive breastfeeding was being practised by 36.1% of mothers of children less than 6 months of age, while 51.4% were predominantly breastfeeding. 2Predominant breastfeeding was defined in accordance with WHO IYCF indicators and allows for infants' consumption of certain liquids in addition to breastmilk, specifically water and water‐based drinks, fruit juice and ritual fluids (WHO 2008). Over half (55.1%, *n* = 162) of mothers of children less than 24 months of age practised bottle feeding.

**Table 4:** Current breastfeeding practices among mothers

	*n*	%
Currently breastfeeding (months)		
0–5	72	83.3
6–11	73	69.9
12–17	72	68.1
18–23	77	23.4
Exclusive breastfeeding*	72	36.1
Predominant breastfeeding*	72	51.4
Continued breastfeeding at 1 year†	52	67.3
Continued breastfeeding at 2 years‡	32	12.5
Bottle feeding (months)		
0–5	72	45.8
6–11	73	67.1
12–17	72	51.4
18–23	77	55.8

* Among children 0–5 months.

† Among children 12–15 months.

‡ Among children 20–23 months.

Forty‐one per cent of all mothers interviewed (*n* = 122) reported providing a breastmilk substitute as a prelacteal feed to their youngest child; 52.5% of mothers who provided a breastmilk substitute as a prelacteal feed reported doing so because they believed that their milk had not yet come in, 26.2% reported that their milk supply was low, and 10.7% reported wanting to start the child on breastmilk substitutes because it would be easier to leave the child to go to work. Over two‐thirds (67.4%) of nonexclusively breastfed children under 6 months were given a breastmilk substitute in the previous day. Sixty‐one per cent were given plain water, and 26.1% were given bottled water. Forty‐three per cent of all children less than 6 months of age had consumed a breastmilk substitute on the day prior to interview. Fourteen per cent of children less that 6 months of age consumed a breastmilk substitute manufactured by Dumex, and 12.5% had consumed a France Bebe product.

Breastmilk substitute consumption was also prevalent among children 6 months of age and older; almost one‐third of children (29.3%, *n* = 65) had consumed this in the previous day. Similar to children below 6 months of age, Dumex and France Bebe products were the most commonly used products among children 6–23 months of age, at 9.0% and 9.5%, respectively. Consumption rates were consistently high across age categories; 39.7% of children aged 6–11 months of age and 24.2% of children aged 12–23 months had consumed a breastmilk substitute in the day prior to interview. Several socioeconomic and demographic characteristics were associated with breastmilk substitute use. Forty‐one per cent of children of mothers who had attended secondary level education or higher had consumed a breastmilk substitute in the prior day, as compared with 21.8% of children of mothers who had only attended primary school or reported no formal education (*P* = 0.001). As compared with children living in households without, children living in houseolds with a refrigerator or a car were more likely to have consumed a breastmilk substitute in the prior day (56.6% vs. 24.3% for refrigerator ownership and 55.0% vs. 26.9% for car ownership; *P* < 0.001 in both cases). Additionally, household television ownership was associated with breastmilk substitute use; 35.2% of mothers who reported owning a television fed their youngest child a breastmilk substitute on the prior day, as compared with 18.2% of mothers whose household did not own a television (*P* = 0.035). Working status of mothers was also found to be borderline significantly associated with use; 42.3% of mothers who reported working outside the home also reported feeding breastmilk substitutes, as compared with 29.6% of mothers who did not work outside the home (*P* = 0.059). Twenty‐nine per cent of mothers who reported themselves as the main caregiver of their youngest child also reported that their child consumed a breastmilk substitute in the day prior to interview, as compared with 50.0% of children of mothers who were not the main caregiver of their youngest child (*P* = 0.008).

Interviewers asked mothers if there were any foods they would like to feed their child if they had more money. Nearly half (45.2%, *n* = 133) of all mothers reported wanting to feed additional foods if they had the financial ability to do so. Breastmilk substitutes were the most commonly reported (15.6%, *n* = 46) food that mothers aspired to buy for their children, closely followed by meat (15.3%, *n* = 45) and fruit (14.3%, *n* = 42). There was no statistical difference in aspirations to feed breastmilk substitutes by age of the child (*P* = 0.841).

Mothers who reported having food aspirations were also asked why they would like to feed these foods if they had enough money. The main reason these 46 mothers reported aspiring to feed a breastmilk substitute to their child was because they believed that there was a direct benefit for the child; 78.3% reported believing that it was healthy for the child, and 32.6% reported believing that it would make the child smart. Reasons related to time availability were less common, with 15.3% of these mothers reporting wanting to feed a breastmilk substitute because they have to go to work and only 5.3% because it would be convenient (more than one reason could be reported).

### Predictors of breastmilk substitute utilisation

Mothers' use of breastmilk substitutes as prelacteal feeds was found to be highly predictive of mothers' current utilisation of these products; 63.5% of mothers who used a breastmilk substitute as a prelacteal feed with their youngest child also reported that this child currently consumed breastmilk substitutes, as compared with 36.5% of mothers who did not use a breastmilk substitute as a prelacteal feed (*P* < 0.001). Exposure to promotion was not found to be significant with mothers' current utilisation of breastmilk substitutes (33.6% of mothers who reported observing a promotion currently fed their child a breastmilk substitute, as compared with 26.8% of moms who did not report observing a promotion, *P* = 0.474). Receiving a recommendation from a health professional to use a breastmilk substitute was also not associated with current use (*P* = 0.874).

Results of multivariate analysis assessing factors associated with current utilisation of breastmilk substitutes are shown in Table 5. Younger children, children of mothers with higher education, children of mothers who worked outside the home and children of mothers who provided a breastmilk substitute as a prelacteal feed were all significantly more likely to have been fed a breastmilk substitute in the day prior to interview. Children of mothers who provided them a breastmilk substitute as a prelacteal feed were 3.9 times more likely to be currently consuming breastmilk substitutes, as compared with children of mothers who did not use breastmilk substitutes as a prelacteal feed (*P* < 0.001).

**Table 5:** Multivariate logistic regression of predictive variables for current utilisation of breastmilk substitutes (*n* = 294)

	Unadjusted		Adjusted	
	*P*‐value	OR (95% CI)	*P*‐value	OR (95% CI)
Child's age	0.007	0.95 (0.91–0.99)	<0.001	0.94 (0.91–0.98)
Maternal educational attainment	<0.001	2.45 (1.45–4.15)	<0.001	2.62 (1.52–4.51)
Mother works outside the home	0.049	1.74 (1.00–3.02)	<0.001	2.26 (1.51–3.40)
Use of breastmilk substitute as prelacteal feed	<0.001	3.91 (2.34–6.54)	<0.001	3.94 (2.44–6.37)

OR, odds ratio; CI, confidence interval.

## Discussion

This study indicates that promotion of breastmilk substitutes in Phnom Penh, Cambodia, is highly prevalent. Phnom Penh mothers frequently reported observing commercial promotions on television and within stores, branding on health facility materials/equipment and recommendations from health professionals to use breast‐milk substitutes. Consumption of breastmilk substitutes was also high among children less than 24 months of age; 43.1% (*n* = 31) of children 0–5 months and 29.3% (*n* = 65) of children 6–23 months had consumed a breastmilk substitute in the day prior to interview. Prelacteal feeding of breastmilk substitutes was also highly prevalent at 41.5% (*n* = 122) of mothers.

The high rate of use of breastmilk substitutes among children under 24 months of age, both as a prelacteal feed and as a continued use during the first 2 years, is a serious concern. The use of breastmilk substitutes negatively impacts exclusive breastfeeding and can shorten the duration of breastfeeding (Sundaram *et al*. 2013), both of which can have wider nutrition and health effects on children and mothers (Stuebe 2009). Findings from this survey show low rates of exclusive breastfeeding of children less than 6 months of age, a low rate of continued breastfeeding at 2 years and high rates of bottle use among children below 24 months. Comparison of national level data indicates that the rate of prelacteal feeding in urban Cambodia has been decreasing, from 56.9% in 2005 to 25.8% in 2010 (NIPH/NIS 2006; NIS 2011); however, findings from this survey indicate that rates in Phnom Penh remain high. In this study, mothers who used a breastmilk substitute as a prelacteal feed with their youngest child were 3.9 times more likely to currently feed this same child breastmilk substitutes, as compared with mothers who did not use these products for prelacteal feeding. Recent secondary analysis of CDHS findings from 2000 to 2010 also indicates the greatest rise in bottle use among the urban poor, a population particularly at risk for use of contaminated water during mixed feeding (Prak *et al*. 2014). In addition to decreased risk of morbidity (Clemens *et al*. 1999; Dewey *et al*. 1995) and mortality (Victora *et al*. 1992; Labbok *et al*. 2004; WHO 2000) for children, the benefits of breastfeeding for mother and child are wide, including reduced risk of obesity (Melnik 2014; Gillman *et al*. 2006), allergies (Kramer 2011; Melnik 2014), asthma (Kim *et al*. 2009) and chronic disease (Owen *et al*. 2006; Martin *et al*. 2005; Owen *et al*. 2002) for children later in life and reduced risk of cancers (Awatef *et al*. 2010; Jordan *et al*. 2012) and diabetes (Liu *et al*. 2010) for mothers. Additionally, a recent study found an association between breastfed children and higher intelligence and educational attainment later in life (Victora *et al*. 2015).

In 2005, Cambodia passed the Sub‐Decree on Marketing of Products for Infant and Young Child Feeding (no. 133) in order to enact the *International Code of Marketing of Breast‐milk Substitutes* (Kingdom of Cambodia 2005) on a national scale. Articles 13–15 of Sub‐Decree 133 specifically cover restrictions on the promotion of breastmilk substitutes and any other food products marketed to children less than 24 months of age within the health system, as well as restricting commercial promotion outside the health system unless prior approval from the Ministry of Health has been obtained. These findings indicate pervasive promotion by manufacturers despite restrictive national law and noncompliance with international guidelines on marketing of breastmilk substitutes.

Various studies have assessed the influence of commercial promotion within the health sector and its impact on breastfeeding patterns, including the impact of free samples or ‘discharge packs’ on rates of exclusive breastfeeding (Rosenberg *et al*. 2008; Frank *et al*. 1987) and duration (Dougherty & Kramer 2006), and exposure to facility‐based advertising and early cessation of breastfeeding (Howard *et al*. 2000). This study found not only that promotion within the Phnom Penh health system exists but also that promotion outside the health system, particularly via television and points of sale, is also prevalent. While many factors influence a mother's breastfeeding decisions and use of breastmilk substitutes, including time, support, familial sway and sociocultural factors, it is likely that promotion of products also plays a substantial role. Mothers' observation of a commercial promotion for breastmilk substitutes was not significantly associated with their youngest child's current utilisation of these products; however, we believe that this lack of significant association is believed to be due to the high (86.1%) prevalence of exposure to promotion, which resulted in an extremely small comparison group of mothers who were not exposed to promotion. Other findings from this survey and follow‐up studies indicate that promotion is influential in Phnom Penh mothers' decisions to use breastmilk substitutes. Among mothers who aspired to feed their child additional foods if they could afford them, the food most desired was breastmilk substitutes because mothers reported that these products were ‘healthy’ for their child and would ‘make them smart’. A review of television commercials aired from September 2013 to September 2014 in Phnom Penh found various commercials advertising breastmilk substitutes. Several commercials contained messages suggesting or implying benefits for children, including ‘If children's immune systems are strong, they can be safe from infectious diseases, and they are strong and grow taller’ and ‘Lactogen 3 for growing and happy children’ (HKI 2014). Among mothers interviewed in this study who aspired to feed breastmilk substitutes, many mentioned the belief that these products would improve the health, growth and intelligence of their child.

Following this study, additional qualitative research was conducted among mothers with children aged 6–23 months in Phnom Penh, both users and nonusers of breastmilk substitutes, to better understand the impact of commercial advertisements on their perceptions of these products. Mothers were shown recordings of advertisements televised in Phnom Penh; the mothers then spontaneously recalled the health and nutrition claims of breastmilk substitutes, including impact on intelligence, growth and development and vitamin/nutrient content claims (HKI 2015). Despite awareness of the benefits of breastfeeding, many mothers who had ever used breastmilk substitutes reported that advertising persuaded them to try the products (HKI 2015). From 2000 to 2010, Cambodia made great strides in improving the rates of early initiation and exclusive breastfeeding (NIS 2011); however, findings from the 2014 CDHS indicate a deterioration in breastfeeding practices (NIS 2015). As the capital and most populous city in Cambodia, Phnom Penh may serve as an influential model for practices among mothers throughout the rest of the country. In addition to reducing the promotion of breastmilk substitutes within Phnom Penh health facilities, there is a great need to combat the pervasive commercial marketing that is occurring in Phnom Penh stores and across Cambodian television channels in order to protect and support breastfeeding nationally.

There are several limitations to this study, mainly related to the fact that this survey was health facility based. The main objective of the study was to assess promotions within Phnom Penh's health system; therefore, interviews were conducted at health facilities. However, this may bias assessment of early breastfeeding practices and utilisation of breastmilk substitutes among the general population of Phnom Penh mothers, as there may be differences between mothers who utilise health care and those who do not. Additionally, we only included public health facilities in this survey; mothers who utilise private health care may have different socioeconomic characteristics than mothers who utilise public health care, which could influence IYCF practices. Specifically, they may be more educated and of a higher income bracket; given that educational attainment was found to be associated with the use of breastmilk substitutes in this study, it may be that our findings are an underestimation of breastmilk substitute utilisation among Phnom Penh mothers because sampling occurred at public health facilities. Finally, the cross‐sectional design of this research limits the ability to establish causality between exposure to promotion and current utilisation of breastmilk substitutes among mothers.

## Source of funding

This study is funded by the Bill & Melinda Gates Foundation.

## Conflicts of interest

The authors have no conflicts of interest to declare.

## Contributions

AP analyzed the data and prepared the manuscript. SH & EZ conceptualized and designed the study, with input from HK. MC oversaw questionnaire development and technology for data collection. KM oversaw data collection. MR oversaw enumerator training. All authors reviewed and provided input on the final article.
